# GABA_A_ receptor subtype selectivity of the proconvulsant rodenticide TETS

**DOI:** 10.1007/s00204-017-2089-4

**Published:** 2017-10-16

**Authors:** Brandon Pressly, Hai M. Nguyen, Heike Wulff

**Affiliations:** Department of Pharmacology, Genome and Biomedical Sciences Facility Room 3502, 451 Health Sciences Drive, School of Medicine, University of California, Davis, CA 95616 USA

**Keywords:** TETS, GABA_A_ receptor, Electrophysiology, Picrotoxinin, Convulsant, Threat agent

## Abstract

The rodenticide tetramethylenedisulfotetramine (TETS) is a potent convulsant (lethal dose in humans 7–10 mg) that is listed as a possible threat agent by the United States Department of Homeland Security. TETS has previously been studied in vivo for toxicity and in vitro in binding assays, with the latter demonstrating it to be a non-competitive antagonist on GABA_A_ receptors. To determine whether TETS exhibits subtype selectivity for a particular GABA_A_ receptor combination, we used whole-cell patch-clamp to determine the potency of TETS on the major synaptic and extrasynaptic GABA_A_ receptors associated with convulsant activity. The active component of picrotoxin, picrotoxinin, was used as a control. While picrotoxinin did not differentiate well between 13 GABA_A_ receptors, TETS exhibited the highest activity on α2β3γ2 (IC_50_ 480 nM, 95% CI 320–640 nM) and α6β3γ2 (IC_50_ 400 nM, 95% CI 290–510 nM). Introducing β1 or β2 subunits into these receptor combinations reduced or abolished TETS sensitivity, suggesting that TETS preferentially affects receptors with α2/β3 or α6/β3 composition. Since α2β3γ2 receptors make up 15–20% of the GABA_A_ receptors in the mammalian CNS, we suggest that α2β3γ2 is probably the most important GABA_A_ receptor for the seizure-inducing activity of TETS.

## Introduction

GABA_A_ receptors are heteropentameric ligand-gated chloride channels that are activated by gamma-aminobutyric acid (GABA), the main inhibitory neurotransmitter in the adult CNS. GABA_A_ receptors have a complex and often somewhat promiscuous pharmacology with numerous orthosteric and allosteric sites that modulate channel function (Krall et al. 2015; Olsen 2015). While GABA_A_ receptor agonists and positive allosteric modulators reduce neuronal excitability and can be used as anxiolytics and anticonvulsants, compounds that inhibit GABA_A_ receptor functions increase neuronal firing and promote seizures. TETS (tetramethylenedisulfotetramine) and picrotoxin are both potent convulsants (Haskell and Voss 1957; Zolkowska et al. 2012) that can cause severe tonic–clonic seizures and are, therefore, considered threat agents by the United States Department of Homeland Security. Both compounds are thought be to work through a similar mechanism; nonselective inhibition of GABA_A_ receptors, yet TETS is 30–100-fold more potent as a convulsant and lethal toxin in the mouse than picrotoxin (Lamanna and Hart 1968; Shandra et al. 1996).

**Fig. 1:**
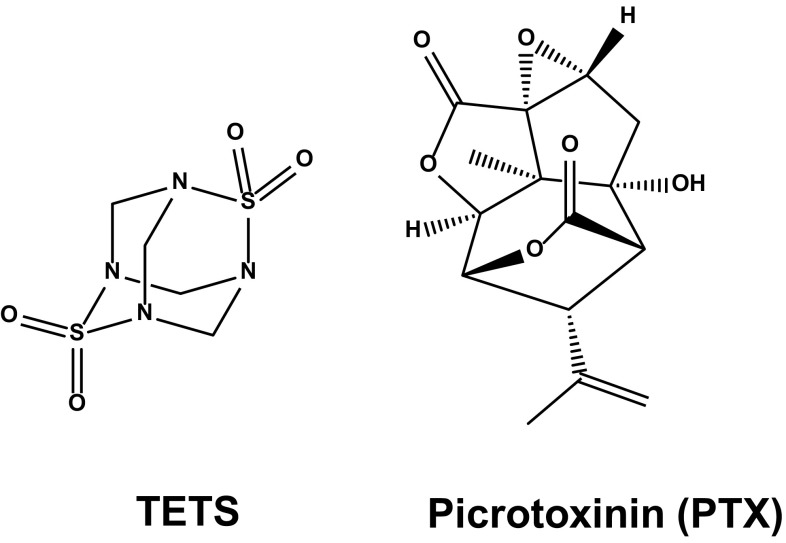
Chemical structures of TETS and picrotoxinin

GABA_A_ receptors exhibit a high degree of structural heterogeneity and exist in multiple subtypes (Olsen and Sieghart 2009), with each subtype being a pentamer assembled from a pool of 19 possible subunits, α1–α6, β1–β3, γ1–γ3, δ, ε, π, θ, and ρ1–ρ3. Based on the fact that TETS and picrotoxin are structurally somewhat similar but distinct (Fig. 1), we wondered if the two agents would exhibit different GABA_A_ receptor subtype specificities. Picrotoxin can be isolated from seeds of the moonseed family and has an extremely bitter taste. It is an equimolar mixture of two tricyclic sesquiterpenes: picrotin and the active component picrotoxinin (Slater and Wilson 1951). TETS has similar physicochemical properties, but is easy to synthesize, tasteless and odorless, and stable in drinking water for months (Knaack et al. 2014). These characteristics make TETS a tangible threat.

TETS was first synthesized in 1933 from sulfamide and formaldehyde (Wood and Battye 1933) and then used as an anti-mold agent for upholstery. Its severe toxicity first became apparent in a German furniture factory where workers accidentally exposed to TETS-impregnated wool suffered from disorientation and seizures (Hagen 1950). TETS was later resynthesized under laboratory conditions and found to be exceptionally poisonous with an extremely low parenteral LD_50_ of 0.1–0.3 mg/kg in rodents (Casida et al. 1976; Haskell and Voss 1957). The United States Forestry Department explored TETS as a rodenticide but eventually abandoned it due to its extreme toxicity, the lethal dose in adult humans being 7–10 mg (Guan et al. 1993), the lack of a viable rescue agent, and its persistence in the environment (Whitlow et al. 2005). TETS is currently banned worldwide, but continues to be easily obtainable and popular as a rodenticide in China, where several mass poisonings with malicious intent have occurred (Whitlow et al. 2005; Zhang et al. 2011). There have also been cases of reported TETS poisonings in the United States with material sourced from China as an indoor rodenticide (Barrueto et al. 2003; Whitlow et al. 2005). The first pharmacological information about TETS was generated by Alfred Haskell and Voss (1957), who tested TETS on organ preparations from frogs, rats, cats, and dogs and found that TETS acted exclusively on the brain without exhibiting any activity on peripheral nerves or muscle. Seizures only terminated with severance below the medulla. The fact that sub-lethal doses of TETS could reverse pentobarbital-induced decreases in blood pressure and respiration in dogs pointed towards a mechanism potentially involving GABA_A_ receptors. TETS was later shown to displace [^35^S]*t*-butylbicyclophosphorothionate ([^35^S]TBPS) binding to rat brain membranes with an IC_50_ of 1 μM (Esser et al. 1991; Squires et al. 1983), and to prevent ^36^Cl^−^ uptake (Ratra et al. 2001) or to inhibit chloride currents (Barnych et al. 2017) through α1β2γ2 GABA_A_ receptors expressed in HEK293 cells with IC_50_ values of 1.3 or 8 μM, respectively. Interestingly, recent work from the Casida laboratory (Zhao et al. 2014) showed that “cold”-TETS displaces [^14^C]TETS from rat brain membranes with a much lower IC_50_ of 80 nM than it displaces another radiolabeled caged convulsant, 4′-ethynyl-4-*n*-[^3^H]propylbicycloorthobenzoate (EBOB), suggesting that TETS is binding to a site that is only partially overlapping with the EBOB or TBPS site on various GABA_A_ receptors or that it might exhibit a specific GABA_A_ receptor subtype selectivity. We here tested the later possibility using patch-clamp electrophysiology in an attempt to find an explanation for the discrepancy between the relatively low potency of TETS in inhibiting GABA_A_ receptors and its high in vivo toxicity (Lamanna and Hart 1968; Zolkowska et al. 2012).

## Materials and methods

### Chemicals

Picrotoxinin (PTX), fipronil, bicuculline, propofol, salicylidene salicylhydrazide, zinc chloride, GABA, dexamethasone, zeocin, and geneticin were purchased from Sigma Aldrich (St. Louis, MO, United States). Diazepam, allopregnanolone, and DS2 (4-chloro-*N*-[2-(2-thienyl)imidazo[1,2-a]pyridin-3-yl]benzamide) were purchased from Tocris Bioscience (Bristol, United Kingdom). TETS was synthesized in the laboratory of Dr. Bruce Hammock, University of California, Davis, CA (Zhao et al. 2014). 10 mM stocks of GABA were made fresh daily using Ringer solution (see below for composition). 10 mM stocks of PTX and TETS were prepared in DMSO and diluted down into Ringer solution immediately before application onto the cell. Both TETS and PTX waste were treated with nitric acid and disposed of using the waste accumulation program at UC Davis.

### Preparation of cells expressing the GABA_A_ receptors

The human GABA_A_ receptors α1, α2, α6, β1, β3, γ2L, and δ and the rat GABA_A_ receptor β2 cloned into pcDNA3.1 expression vectors were a gift from Dr. Robert L. Macdonald, Vanderbilt University, Nashville, TN. The human GABA_A_ receptor α4 cloned into a pcDNA3.1 expression vector was a gift from Dr. Richard Olson, University of California, Los Angeles, CA. The human GABA_A_ receptor γ1 cloned into a pcDNA3.1 expression vector and a Lt-K cell line stably expressing α4β3δ (Mortensen et al. 2010) were gifts from Dr. Trevor Smart, University College London, United Kingdom. L929 cells, a mouse fibroblast cell line (CCL-1), were obtained from ATCC (American Type Culture Collection, Manassas, VA, United States), and were used for expressing all GABA_A_ receptors with the exception of the receptor combination α6β3δ for which COS-7 cells (ATCC CRL-1651) were used to increase transfection efficacy and expression. L929, Lt-K, and COS-7 cells were cultured in Dulbecco’s modified Eagle’s medium (Lonza, Basel, Switzerland) supplemented with 10% fetal bovine serum, 100 U/mL penicillin and 100 mg/mL streptomycin (Invitrogen, ThermoFisher, Grand Island, NY, United States) and maintained in humidified 95% air and 5% CO_2_ air at 37 °C. The Lt-K cell line expressing α4β3δ was cultured with 1 mg/mL geneticin and 0.2 mg/mL of zeocin to maintain selection pressure. Two days before experiments, 1 μM dexamethasone was added to the media to induce α4 and β3 expressions (Mortensen et al. 2010). L929 or COS-7 cells were transfected using FuGENE 6 (ThermoFisher, Grand Island, NY, United States) transfection reagent in Opti-MEM^®^ reduced serum medium (Life Technologies, Benicia, CA, United States) with an equal amount of each of the subunits (1:1:1) in combination with green fluorescent protein (GFP) expressed from the pEGFP-C1 vector (Invitrogen). The ratio of total cDNA to transfection reagent was 2:1. 48 h post-transfection, and cells were detached by trypsinization, washed, and plated onto poly-l-lysine-coated glass coverslips. Transfected cells were identified as GFP-expressing cells, using an epifluorescence microscope for electrophysiological whole-cell voltage-clamp studies. Correct subunit assembly was tested with a battery of GABA_A_ receptor positive allosteric modulators and inhibitors (Table 1).

**Table 1:** Pharmacological signature of expressed GABA_A_ receptor isoforms

GABA_A_ isoform	Sensitivity to positive allosteric modulators	Sensitivity to inhibitors
α1β12γL	Diazepam^+^, Propofol^+^, Allopregnanolone^+^	SAS^+^, Bicuculline^+^, Fipronil^+^
α1β2γ2L	Diazepam^+^, Propofol^+^, Allopregnanolone^+^	Fipronil^+^
α1β3γ2L	Diazepam^+^, Propofol^+^, Allopregnanolone^+^	Fipronil^+^
α2β2γ2L	Diazepam^+^, Propofol^+^	Fipronil^+^
α2β3γ2L	Diazepam^+^, Propofol^+^, Allopregnanolone^+^	SAS^neg^, Bicuculline^+^, Fipronil^+^, Zn^2+neg^
α2β3	Diazepam^neg^, Propofol^+^,	Fipronil^+^
α4β3γ2L	Diazepam^neg^, Propofol^+^,	Fipronil^+^
α4β3δ	Diazepam^neg^, Allopregnanolone^+^, DS2^+^	Fipronil^+^
α6β1γ2L	Diazepam^neg^	SAS^+^, Bicuculline^+^, Fipronil^+^
α6β2γ2L	Diazepam^neg^, Propofol^+^	Fipronil^+^
α6β3γ2L	Diazepam^neg^, Propofol^+^, Allopregnanolone^+^	SAS^neg^, Bicuculline^+^, Fipronil^+^, Zn^2+neg^
α6β3	ND	Zn^2+^ positive
α6β3γ1	Diazepam^neg^, Propofol^+^	Fipronil^+^
α6β3δ	Diazepam^neg^, DS2^+^	Fipronil^+^

Sensitivity to 10 μM diazepam, 250 nM Allopregnanolone, 100 μM Propofol, 50 μM DS2 (4-chloro-*N*-[2-(2-thienyl)imidazo[1,2-a]pyridin-3-yl]benzamide), 10 μM Fipronil, 100 μM Bicuculline, 10 μM SAS (salicylidene salicylhydrazide) and 10 μM ZnCl_2_ was tested on 3–10 cells per subunit combination

*ND* Not determined

### Electrophysiological recordings

Whole-cell voltage-clamp experiments were performed at room temperature with an EPC-10 HEKA amplifier (HEKA Elektronik, Lambrecht, Germany). Cells were bathed in Ringer solution consisting of 160 mM NaCl, 4.5 mM KCl, 1 mM MgCl_2_, 2 mM CaCl_2_, 10 mM HEPES, pH 7.4, 311 mOsm. Recording electrodes were pulled from soda lime glass micro-haematocrit tubes (Kimble Chase, Rochester, NY, United States) and fire-polished to resistances of 1.8–2.8 MΩ for voltage-clamp. Electrodes were filled with an internal solution consisting of 154 mM KCl, 2 mM CaCl_2_, 1 mM MgCl_2_, 10 mM HEPES, and 10 mM EGTA with pH 7.2 and 305 mOsm. Cells were voltage-clamped at − 80 mV and control currents were recorded under the application of varying GABA concentrations for 5 s followed by a 55 s wash with external Ringer solution using a gravity-fed fast perfusion system (VC^3^8 system, ALA Scientific, Farmingdale, NY, United States).

The GABA concentration–response relationships were determined by testing increasing concentrations of GABA and normalizing the GABA-induced peak currents to the peak currents induced by a maximal concentration of GABA. The normalized current responses were subsequently fitted using the Hill equation to determine EC_50_ and EC_90_.

For the TETS and PTX concentration–response curves, test solutions were freshly prepared immediately before application onto cells. A near maximal concentration of GABA (GABA EC_90_) was applied for 5 s and then washed out for 55 s. TETS or PTX was then perfused in a volume of 3–5 mL directly into the bath through a side port on the perfusion chamber and cells incubated with the inhibitor for 3 min before a 5 s pulse of GABA was applied to the patch-clamped cell. The convulsant was then washed out and another control GABA pulse applied. One cell was used per concentration of the convulsant. For analysis of receptor blockade, the area under the current curve (AUC_Max_) was determined for the control (EC_90_ GABA) and AUC_Ex_ after exposure:

$$\frac{{{\text{AUC}}_{\text{Ex}} }}{{{\text{AUC}}_{\text{Max}} }} \times 100 = \% {\text{Blocked}} .$$

For competition experiments, drug was applied for 5 s followed by 55 s of wash with Ringer. The cells were then exposed to GABA or GABA plus the convulsant (TETS or PTX) without any pre-incubation. GABA-induced responses in the presence and absence of convulsant were quantified by analyzing the peak response.

Data analysis was performed using the Excel (Microsoft) and Origin 9.1 (OriginLab Corporation, Northampton, MA, United States) software. Data fitting to the Hill equation to obtain EC_50_ or IC50 values were also performed using Origin 9.1. Individual data points are presented as mean ± SD from 3 to 8 independent recordings. EC_50_ and IC_50_ values are presented with 95% confidence intervals.

## Results

### GABA concentration responses

**Fig. 2:**
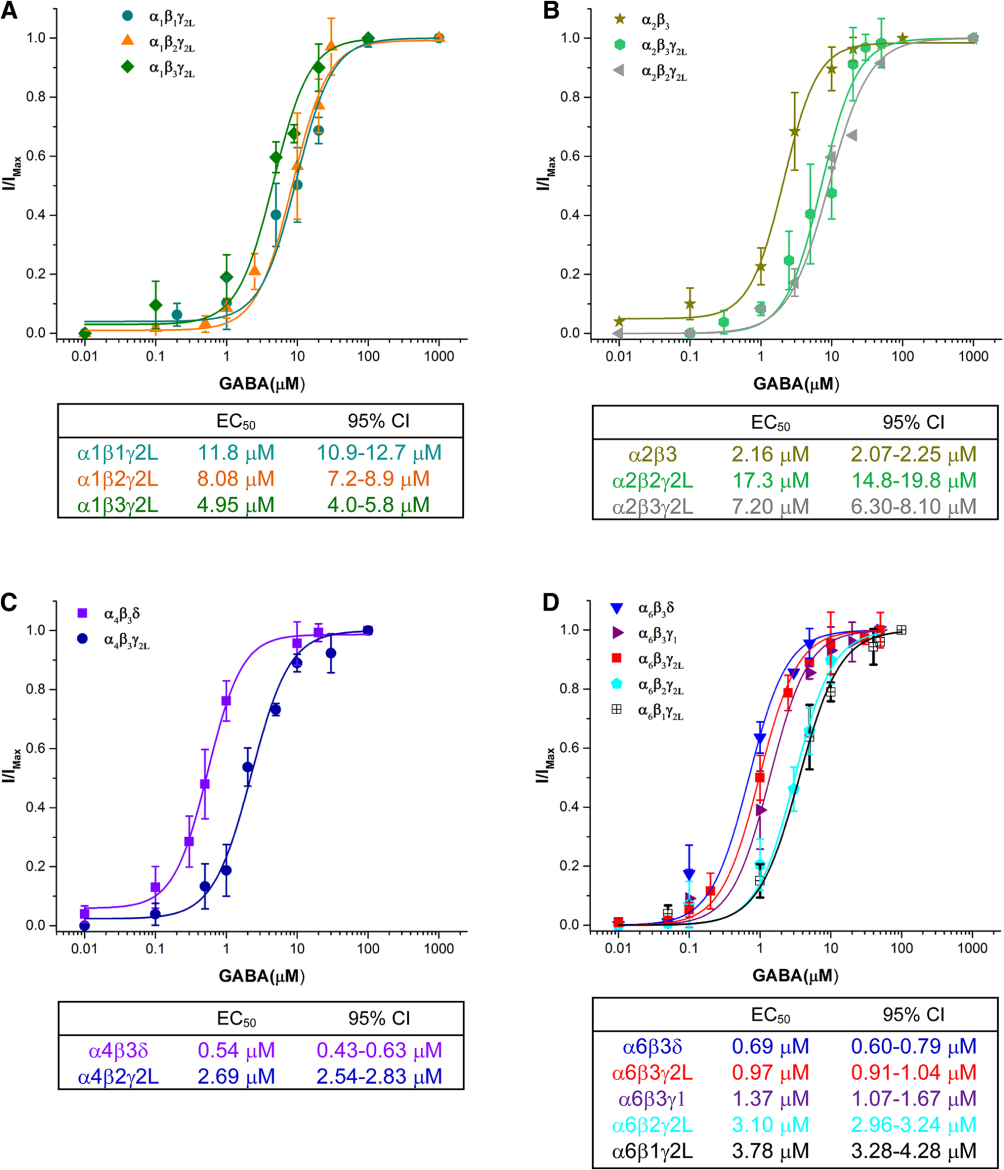
GABA concentration–response curves for α1 (**a**), α2 (**b**), α4 (**c**), and α6 (**d**) containing GABA_A_ receptor combinations. Individual data points are presented as mean ± SD from 7–20 independent recordings. EC_50_ values are presented with 95% confidence intervals

Before studying TETS activity, we first obtained GABA concentration–response curves for 13 synaptic and extrasynaptic GABA_A_ receptor isoforms transiently or stably expressed in L929 cells, a mouse fibroblast cell line that has been used to express GABA_A_ receptors since the early 1990s (Angelotti et al. 1993). Correct subunit assembly and γ2L or δ incorporation were confirmed with a battery of GABA_A_ receptor probes consisting of the positive allosteric modulators diazepam, propofol, allopregnanolone, and DS2 and the inhibitors fibronil, bicuculline, salicylidene salicylhydrazide, and zinc chloride (Table 1). For both the exemplary synaptic GABA_A_ receptors α1β1γ2L, α1β2γ2L, α1β3γ2L (Fig. 2a), α2β3, α2β2γ2L, and α2β3γ2L (Fig. 2b) and the exemplary extrasynaptic GABA_A_ receptors α4β3δ, α4β3γ2L (Fig. 2c), α6β3δ, α6β3γ1, α6β2γ2L, α6β3γ2L, and α6β1γ2L (Fig. 2d), we determined GABA EC_50_ and EC_90_ values to choose the most appropriate GABA concentrations for subsequently evaluating TETS and PTX effects on each of these receptors. When comparing our EC_50_ values to the previous work in the field, we found that most of our results are in good agreement with data obtained by Mortson et al. (2011), who transiently expressed various synaptic and extrasynaptic GABA_A_ receptors in HEK cells and reported a very similar overall ranking of GABA sensitivity. However, we observed a few differences. In our hands, α6 containing combinations were generally 2–3-fold less sensitive to GABA than previously reported. For example, we obtained an EC_50_ of 0.69 μM for α6β3δ (Fig. 2d), whereas Mortenson et al. reported EC_50_ of 0.17 μM for this same subunit combination (Mortensen et al. 2011). Despite this difference in sensitivity, we observed the expected pharmacological responses, specifically, no response to diazepam but a pronounced response to DS2 (Table 1).

### Importance of the β subunit in TETS activity

**Fig. 3:**
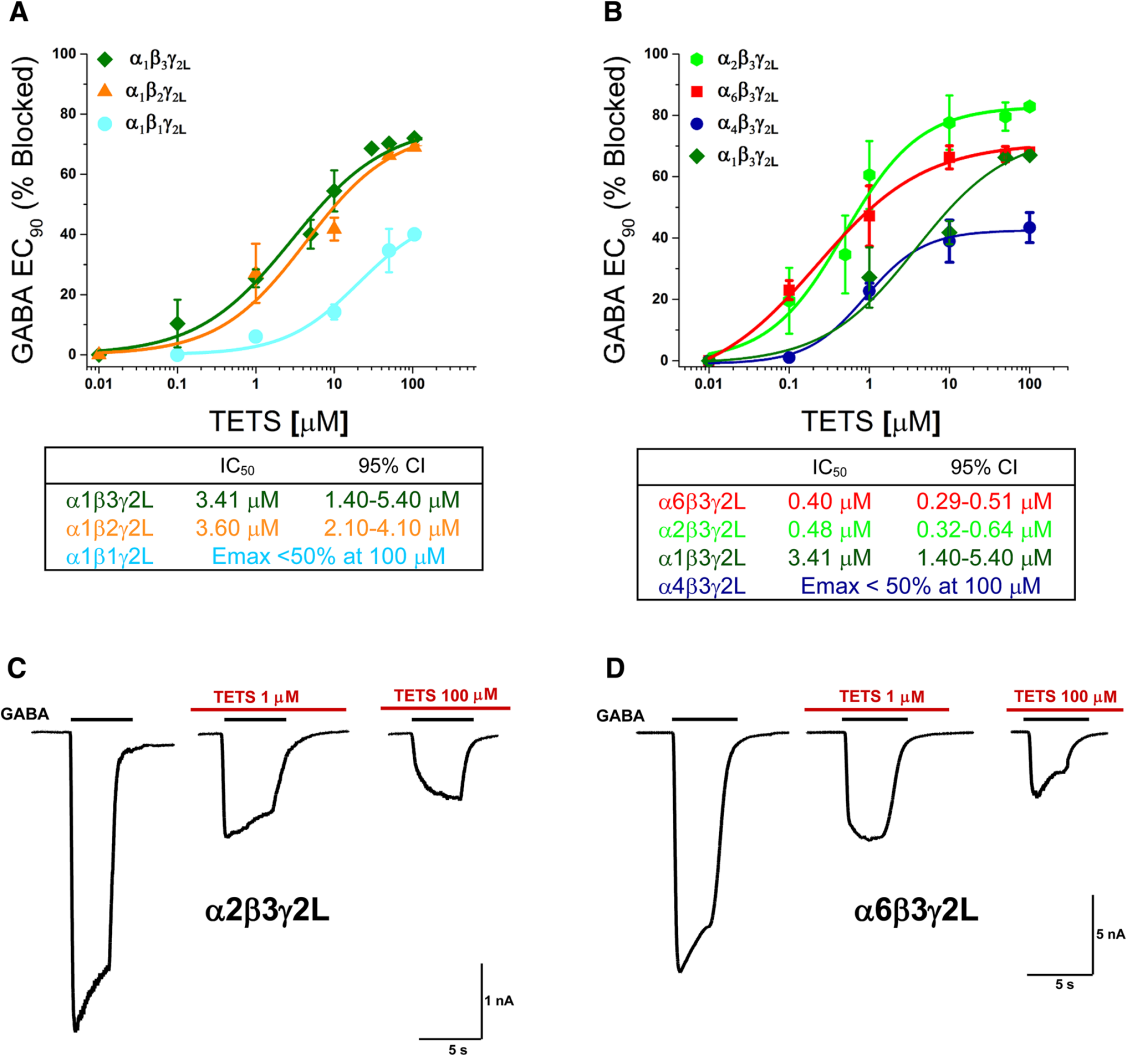
**a** Concentration–response curves comparing TETS inhibition of different β subunits in the α1βXγ2L combination. **b** Comparison of TETS inhibition of α1, α2, α4, and α6 subunits in αXβ3γ2L containing GABA_A_ receptor combinations. Individual data points are presented as mean ± SD from 3–8 independent recordings. EC_50_ values are presented with 95% confidence intervals. Example traces showing TETS inhibition of α2β3γ2L (**c**) and α6β3γ2L (**d**) receptors. EC_90_ GABA was applied first alone as control and then in the presence of 1 or 100 μM of TETS

Based on molecular dynamics simulations using a homology model of the pore region of the α1β2γ2 GABA_A_ receptor, TETS has been hypothesized to bind in the pore lumen by hydrogen bonding with two α1–M2 segments and one γ2–M2 segment without making any contacts with the β subunit (Zhao et al. 2014). In support of this hypothesis, the β3 homopentamer has been shown to not bind TETS even at concentrations greater than 10 μM (Ratra et al. 2001). To determine if the β subunit is really not important for TETS activity, as suggested by these studies, we tested all three β subunits, β1, β2, and β3, in combination with α1 and γ2 (Fig. 3a). We started with α1β2γ2, since it is the most abundant subunit combination, constituting ~ 60% of GABA_A_ receptors in the mammalian brain (Rudolph and Knoflach 2011; Sur et al. 2001). GABA-induced chloride current at GABA EC_90_ through this subunit exhibited a moderate sensitivity to inhibition by TETS (IC_50_ 3.6 μM). Following pre-incubation with different TETS concentrations, block developed quickly, and TETS inhibition was fully reversible on washout; however, even concentrations as high as 100 μM could not fully block the current and only achieved a maximal inhibition of ~ 70%. The remaining GABA-induced chloride current could be blocked by 100 μM of bicuculline or 10 μM of fipronil (data not shown). To determine the significance of changing the β subunit, we replaced it with both β1 and β3. While α1β1γ2 was not very sensitive to TETS (maximal block < 50% at 100 μM), α1β3γ2 was indistinguishable from the β2 containing subunit combination in terms of TETS potency and maximal efficacy (Fig. 3a). We, therefore, concluded that both β2 and β3 subunits can modulate TETS binding.

### Understanding the significance of the α subunit in TETS activity on GABA_A_ receptors

We next investigated the role of the α subunit in TETS activity by exchanging α1 for α2, α4, and α6 in the αXβ3γ2L combination (Fig. 3b). We chose this combination rather than the αXβ2γ2L combination, because the β3 we are using for this work is human, whereas β2 is a rat clone. We did not investigate α5, because this subunit is not thought to be involved in seizure generation, but rather plays a role in learning and memory (Collinson et al. 2002; Rudolph and Mohler 2004). Out of the tested subunit combinations, α2β3γ2L (IC_50_ = 480 nM with a maximal block of ~ 80%) and α6β3γ2L (IC_50_ = 400 nM with a maximal block of ~ 75%) showed the highest sensitivity to TETS (Fig. 3b), while α1β3γ2L was roughly tenfold less sensitive (α1β3γ2L: IC_50_ = 3.6 μM with a maximal block of ~ 75%). On the α4β3γ2L combination, even 100 μM of TETS could not achieve more than 45% of inhibition, making this subunit combination the least sensitive.

### Does the β subunit affect the TETS sensitivity of α2 and α6?

**Fig. 4:**
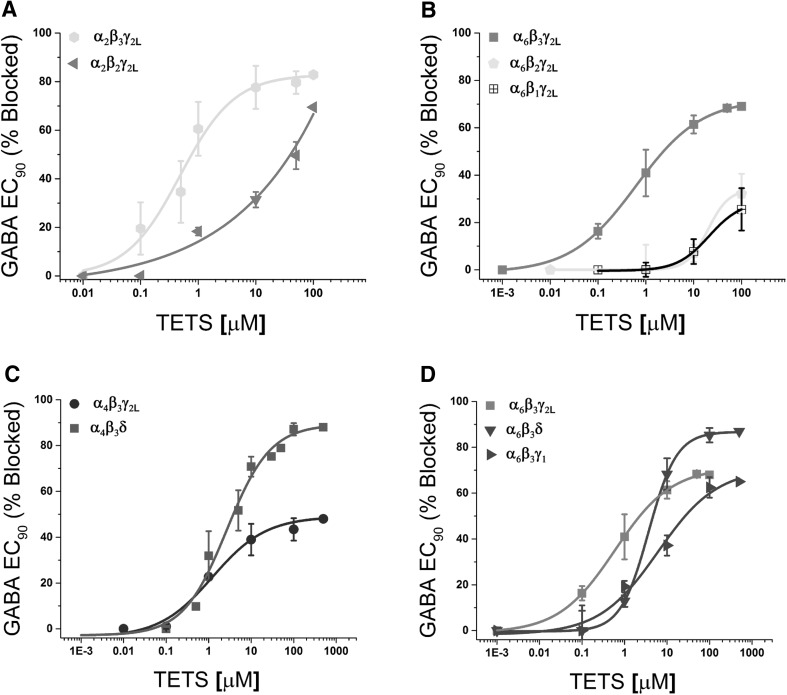
**a** Concentration–response curves for TETS inhibition of currents evoked by EC_90_ GABA for α2βxγ2L receptors to determine the changes induced by exchanging β2 for β3. The concentration response curve of TETS for α2β2γ2L (IC_50_ 12.2 μM, 95% CI 10.5–15.8 μM, *E*_max_ ~ 65%) was right-shifted in comparison with α2β3γ2L (IC_50_ 480 nM, 95% CI 10.5–15.8 nM, *E*_max_ ~ 80%). **b** Both the α6β1γ2L receptor (IC_50_ > 20 μM, *E*_max_ ~ 25%) and the α6β2γ2L receptor (IC_50_ 20 μM, 95% CI 18.3–21.7 μM, *E*_max_ ~ 35%) showed a significantly reduced response to TETS. The α6β3γ2L receptor (IC_50_ 400 nM, 95% CI 290–510 nM, *E*_max_ ~ 70%) is highly sensitive to TETS as shown previously in Fig. 3. **c** α4β3γ2L receptor (IC_50_ 1.82 μM, 95% CI 1.02–2.62 μM, *E*_max_ ~ 46%, *n*_*H*_ = 0.7) showed a reduced *E*_max_ for TETS inhibition, while the α4β3δ receptor (IC_50_ 3 μM, 95% CI 1.36–3.96 μM, *E*_max_ ~ 85%, *n*_*H*_ = 1.2) showed an increased Hill coefficient. **d** In α6 containing receptors (α6β3γ2L: IC_50_ 400 nM, 95% CI 0.29–0.51 nM *E*_max_ ~ 70% as previously shown), introduction of a δ subunit increased *E*_max_ and reduced potency (α6β3δ: IC_50_ 3.69 μM, 95% CI 3.18–4.2 μM, *E*_max_ ~ 85%), while replacement of γ2L with γ1 right-shifted the concentration response curve (α6β3γ1: IC_50_ 6.81 μM, 95% CI 3.9–9.7 μM, *E*_max_ ~ 65%). Individual data points are presented as mean ± SD from 3–9 independent recordings. EC_50_ values are presented with 95% confidence intervals. Please note that the α4β3δ receptor combination was stably expressed in Lt-K cells, while the α6β3δ combination was transiently expressed in COS-7 cells

We had seen that there was no difference in TETS sensitivity between β2 and β3 when these subunits were expressed with α1 (Fig. 3a). It was important to determine if this was also true for the more sensitive α2 and α6 subunits and we, therefore, next co-expressed these two subunits with β2 instead of β3. In both combinations, TETS clearly showed a preference for β3 containing subunit combinations over β2 containing combinations (Fig. 4a, b). For the α2βXγ2L combination, replacing β3 with β2 shifted the TETS concentration–response curve significantly to the right, and increased IC_50_ from 400 nM to 12.2 μM (Fig. 4a). For the α6βXγ2L combination, the effect was even more dramatic, and in the presence of β2, even 100 μM of TETS no longer achieved more than 40% inhibition of GABA-induced chloride current (Fig. 4b). Replacing β3 with β1 further reduced TETS activity with 100 μM of TETS only achieving 25% inhibition of current through α6β1γ2L (Fig. 4b). Based on these results, we conclude that the presence of an α2 or α6 subunit and a β3 subunit is necessary for TETS to inhibit chloride currents through GABA_A_ receptors with high potency and efficacy.

### Significance of the γ and δ subunits in TETS activity

We next tested if δ could possibly play a role in TETS activity, and, therefore, determined its potency for blocking currents through the extrasynaptic α4β3δ GABA_A_ receptor stably expressed in Lt-K cells and the α6β3δ receptor transiently transfected into COS-7 cells. We used COS-7 cells for these experiments, because the α6β3δ combination did not express well in L929 cells (peak currents < 200 pA). We chose not to try and generate the α2β3δ GABA_A_ receptor, since there seems to be no evidence for the existence of this subunit combination in the literature (Olsen and Sieghart 2008, 2009). Interestingly, in the two δ containing subunit combinations, we investigated the presence of a δ subunit increased the maximal efficacy of TETS inhibition when compared with the γ2L containing GABA_A_ receptor combinations, and also changed the Hill coefficient (Fig. 4c, d). However, it should be noted here that the two δ containing subunit combinations in our study, α4β3δ and α6β3δ, were expressed in two different cell lines, Lt-K and COS-7, which could have impacted our results.

Overall, α4 containing receptors are not very sensitive to TETS (α4β3γ2L: IC_50_ = 1.82 μM, *E*_max_ = 46%, *n*_*H*_ = 0.7), as previously shown in Fig. 3, but TETS inhibited the δ containing α4β3δ receptor with an increased efficacy (IC_50_ = 2.66 μM, *E*_max_ = 85%; Fig. 4c) and a steeper Hill coefficient (*n*_*H*_ = 1.2). The same effect was observed in the more TETS-sensitive α6 combination (Fig. 4d). While α6β3δ was less sensitive (IC_50_ = 3.69 μM) to TETS inhibition than the α6β3γ2L receptor (IC_50_ = 400 nM, *E*_max_ = 64%, *n*_*H*_ = 0.7), TETS displayed a greater efficacy (*E*_max_ = 85%) and a steeper Hill coefficient (*n*_*H*_ = 1.3) for α6β3δ (Fig. 4d). In contrast, exchanging γ2 for γ1 “simply” shifted the TETS concentration–response curve to the right and reduced potency, but maintained efficacy (α6β3γ1: IC_50_ = 6.81 μM, *E*_max_ = 60%). We further tested α2β3 to see how removing the γ subunit would change TETS sensitivity and found that the absence of a γ subunit in the receptor pentamer lowered both the potency and efficacy of TETS (Table 2).

**Table 2:** IC_50_ values for TETS and PTX

GABA_A_ isoform	TETS (μM)	95% CI	PTX (μM)	95% CI
α1β1γ2L	> 20	–	> 30	–
α1β2γ2L	3.60	2.1–4.1	7.98	6.58–9.38
α1β3γ2L	3.41	1.4–5.4	3.7	2.1–5.3
α2β2γ2L	12.2	10.5–15.8	11	8.6–13.5
α2β3γ2L	0.48	0.32–0.64	7.5	5.2–9.9
α2β3	3.37	2.77–3.97	1.98	0.67–3.29
α4β3γ2L	1.82	1.02–2.62	3.0	1.7–4.3
α4β3δ	2.66	1.36–3.96	ND	ND
α6β1γ2L	> 20	–	ND	ND
α6β2γ2L	20	18.3–21.7	26.9	20.3–33.2
α6β3γ2L	0.40	0.29–0.51	5.8	4.2–7.4
α6β3γ1	6.81	3.9–9.7	ND	ND
α6β3δ	3.69	3.18–4.2	ND	ND

*ND* Not determined

### Picrotoxinin shows no GABA_A_ receptor subtype selectivity

**Fig. 5:**
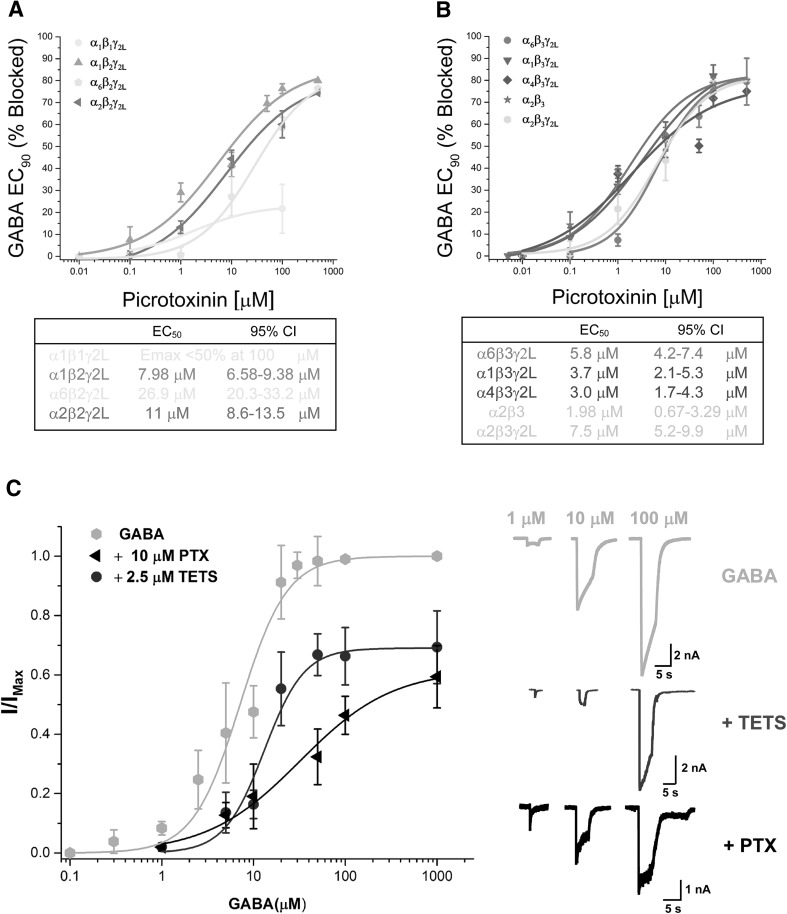
Concentration-response curves for PTX inhibition of β1/β2 (**a**) and β3 (**b**) containing GABA_A_ receptor combinations. **c** Effect of TETS and PTX are roughly IC_80_ concentrations on the GABA concentration response curve of the α2β3γ2L receptor. Individual data points are presented as mean ± SD 3–8 independent recordings. EC_50_ values are presented with 95% confidence intervals

As a control, we tested picrotoxinin (PTX), which is a widely used GABA_A_ receptor inhibitor also classified as a threat agent on the same receptor subtypes (Table 2 and Fig. 5). As expected based on data in the published literature, PTX is a relatively nonselective GABA_A_ receptor inhibitor that blocks GABA-induced Cl^−^ currents through most GABA_A_ receptors with IC_50_s in 2–8 μM range and *E*_max_ of ~ 80% current inhibition. Similar to TETS, the presence of a β1 subunit abolishes PTX sensitivity, since α1β1γ2L is insensitive to PTX, in contrast to various other β2 (Fig. 5a) or β3 (Fig. 5b) containing GABA_A_ receptor combinations, which are all inhibited by PTX (Table 2).

### TETS is a non-competitive GABA_A_ receptor inhibitor

Finally, to start probing the mechanism of action of TETS on the α2β3γ2L receptor, we tested the effect of 2.5 μM TETS, which corresponds to IC_80_ on this receptor subtype, on the GABA concentration–response curve (Fig. 5c). As expected from the previously performed binding experiments (Zhao et al. 2014), TETS depressed the maximum GABA response elicited at 50 or 100 μM GABA and could not be competed off when the GABA concentration was increased to 1 mM, demonstrating that TETS is a non-competitive inhibitor similar to PTX, which was also tested at IC_80_ for comparison. PTX at 10 μM produced a similar depression of the GABA *E*_max_ and was also not able to shift the GABA concentration–response curve fully to the right (Fig. 5c). Similar results were obtained with the other highly TETS-sensitive GABA receptor combination, α6β3γ2L (data not shown).

## Discussion

The rodenticide TETS has previously been primarily studied in animal models to assess its convulsant activity and toxicity or in binding assays, which demonstrated that TETS acts as a non-competitive GABA_A_ receptor inhibitor. However, so far, only very limited electrophysiological information is available for this highly toxic threat agent. In 1975, Bowery et al. demonstrated that TETS could reverse the effects of GABA on isolated superior cervical rat ganglions at concentrations between 10 and 100 μM (Bowery et al. 1975), while a group in the former Soviet Union showed in 1989 that 5 μM of TETS increased the excitability of hippocampal slices (Pervukhin et al. 1989). A more recent paper from our own group describing the synthesis of TETS-related haptens for the development of an ELISA assay to detect TETS (Vasylieva et al. 2017) reported that TETS inhibited chloride currents through α1β2γ2 GABA_A_ receptors expressed in HEK293 cells with IC_50_ of 8 μM (Barnych et al. 2017). Since we were somewhat surprised by this relatively low potency in electrophysiological assays considering TETS has a reported LD_50_ of 0.1 mg/kg (Casida et al. 1976; Haskell and Voss 1957), we here investigated the GABA_A_ receptor subtype selectivity of TETS using whole-cell patch-clamp electrophysiology. One observation that made us suspect that TETS might exhibit subtype selectivity was a report from the Casida laboratory that unlabeled TETS displaced [^14^C]TETS from rat brain membranes with IC_50_ of 80 nM (Zhao et al. 2014), which is much lower than IC_50_s usually reported for TETS displacement of other radiolabeled GABA_A_ receptor antagonists such as EBOB (Zhao et al. 2014) or TBPS (Esser et al. 1991; Squires et al. 1983). Another report suggests that the possibility of a higher affinity target was Ca^2+^ dynamics measurements in mouse hippocampal neuronal cultures which develop spontaneous network activity after about 2 weeks in culture (Cao et al. 2012). TETS visibly altered Ca^2+^ dynamics in these networks at submicromolar concentrations, although the reported EC_50_s for altering the frequency and amplitude of the Ca^2+^-induced fluorescence changes in the assay were the 1–2 μM range (Cao et al. 2012).

Here, we identified two GABA_A_ receptor subtypes that are sensitive to TETS at submicromolar concentrations: α2β3γ2 with IC_50_ of 480 nM and α6β3γ2 with IC_50_ of 400 nM. Of these two receptor subtypes, both of which are among the 11 GABA_A_ receptors conclusively identified as native receptors (Olsen and Sieghart 2008), α2β3γ2 is probably the more important receptor for the seizure-inducing activity of TETS, since α2β3γ2 receptors make up 15–20% of the GABA_A_ receptors in the mammalian CNS (Fritschy and Mohler 1995; Pirker et al. 2000; Rudolph and Knoflach 2011) and α2 containing GABA_A_ receptors have been shown to significantly contribute to the anticonvulsant actions of diazepam (Fradley et al. 2007). In contrast, receptors with the α6β2/3γ2 composition constitute less than 5% of the GABA_A_ receptors (Mohler et al. 2002; Rudolph and Knoflach 2011) and are largely restricted to the cerebellum (Jones et al. 1997). One short coming of our study is that we only used the long splice variant of the γ2 subunit, γ2L, and not the short γ2s subunit, which is known to be expressed throughout the mammalian CNS in similar proportions and often co-localizes with γ2L in the same receptor complexes (Khan et al. 1994).

TETS differs from the less selective PTX in showing preference for α2 and α6 over α1 and α4 when expressed in combination with β3 and γ2. Introducing a β1 subunit into the GABA_A_ receptor heteropentamer dramatically decreases both TETS and PTX potency and efficacy, suggesting that the presence of an α/β1 interface disrupts binding for both compounds. In contrast, exchanging β3 for β2 or γ2 for the less commonly found γ1 reduces TETS activity by roughly tenfold (Table 2). However, β3 alone is not sufficient to generate a TETS binding site, since homopentameric β3 GABA_A_ receptors have been shown to not bind TETS at concentrations of up to 10 μM, while they bind PTX with an affinity of 32 nM (Ratra et al. 2001). It is interesting that the presence of a β3 subunit in the preferred α2/β3 or α6/β3 combination plays such a large role in TETS action on the GABA_A_ receptor. Of these two β subunits, β2 and β3, that are able to participate in TETS binding, the more sensitive β3 subunit is probably more important for the proconvulsant activity of TETS. Mice lacking the β3 subunit display features reminiscent of Angelman syndrome in humans including abnormal EEG with interictal spikes and slowing, seizures, hyperactivity, impaired learning, and memory and repetitive behavior (DeLorey et al. 1998; Handforth et al. 2005). In contrast, mice lacking β2 do not exhibit spontaneous seizures, but are less susceptible to the hypnotic actions of ethanol and the effects of benzodiazepines (Blednov et al. 2003; Sur et al. 2001).

While TETS and PTX thus differ in their GABA_A_ receptor subtype selectivity, with TETS most likely exerting its major action on α2β3γ2 receptors and while PTX does not demonstrate any significant receptor selectivity, we hesitate to use this selectivity as an explanation for the greater in vivo toxicity of TETS, since the differences in potency could possibly also be pharmacokinetic in nature. When administered intraperitoneally in mice, TETS induces clonic seizures with ED_50_ of 0.14 mg/kg and is lethal at a dose of 0.3 mg/kg, which is roughly 40 times more potent than picrotoxin (Zolkowska et al. 2012). However, when TETS and picrotoxin (which in this case contained only 50% of the active picrotoxinin) were administered intraventricularly, they were found to be equipotent in their ability to induce convulsions (Zolkowska et al. 2012), suggesting that the higher potency of TETS when administered systemically could be due to brain penetration or brain uptake than PTX. Another possibility is of course that TETS has additional actions on other receptors than GABA_A_ receptors that promote seizures and convulsions.

Both TETS and PTX failed to fully block GABA-induced chloride currents through all tested GABA_A_ receptor subunit combinations under our experimental conditions in which we elicited near maximal currents using GABA EC_90_ concentrations for each receptor, and could typically only achieve 70–85% of block. It is of course possible that we could have achieved some additional block by applying millimolar TETS or PTX concentrations, but we chose to not test concentrations higher than 100 μM of TETS on most receptors, since we regard these high concentrations as physiologically unrealistic. However, it should be mentioned here that the remaining current could be blocked by bicuculline (100 μM) or fipronil (10 μM). Out of these two compounds, bicuculline is known to competitively inhibit GABA binding at the orthosteric site, although it also seems to have additional allosteric actions (Johnston 2013), while the phenylpyrazole fipronil has been shown to bind to the same site as picrotoxinin and several polychlorocycloalkane insecticides in radioligand binding assays on the homopentameric β3 receptor (Chen et al. 2006). This of course raises the question of the TETS binding site, which based on the non-competitive nature of its inhibition, and the observations that TETS displaces pore blockers like [^35^S]TBPS (Esser et al. 1991; Squires et al. 1983) and the cage convulsant EBOB (Ratra et al. 2001; Zhao et al. 2014) is most likely located in the pore region of the channel, where TETS has been predicted by molecular dynamics simulations to hydrogen bond with two α1–M2 segments and one γ2–M2 segment in a homology model of the α1β2γ2 GABA_A_ receptor. This model (Zhao et al. 2014), which was based on the crystal structure of the homopentameric *Caenorhabditis elegans* glutamate-gated chloride channel (Hibbs and Gouaux 2011), and which has never been probed by mutagenesis, showed TETS making no contacts with the β subunit, thus, apparently providing an explanation for why the α1β2γ2 GABA_A_ receptor is sensitive to TETS, while the β3 homopentamer is not (Ratra et al. 2001). This molecular model should be re-examined in light of our findings that TETS shows the highest potency for blocking chloride current through GABA_A_ receptors containing α2 or α6 as well as β3 subunits. Ideally, the TETS binding site should be mapped through a combination of site-directed mutagenesis and molecular modeling using new homology models of the α2β3γ2, α2β2γ2 and the α1β2γ2 GABA_A_ receptors based on the more recently crystallized human β3 homopentamer (Miller and Aricescu 2014).

## Abbreviations

AUC: Area under the curve
CI: Confidence interval
DMSO: Dimethylsulfoxide
EBOB: 4′-Ethynyl-4-*n*-propylbicycloorthobenzoate
EC_50_: Concentration producing 50% effect
*E*_max_: Maximal effect
GABA: Gamma-aminobutyric acid
GFP: Green fluorescent protein
IC_50_: Concentration producing 50% inhibition
*n*_*H*_: Hill coefficient
PTX: Picrotoxinin
SD: Standard deviation
TETS: Tetramethylenedisulfotetramine
TBPS: *t*-Butylbicyclophosphorothionate
